# Bone Graft Packing and Its Association with Bone Regeneration in Maxillary Sinus Floor Augmentations: Histomorphometric Analysis of Human Biopsies

**DOI:** 10.3390/biology11101431

**Published:** 2022-09-30

**Authors:** Karoline Maria Reich, Florian Beck, Patrick Heimel, Stefan Lettner, Heinz Redl, Christian Ulm, Stefan Tangl

**Affiliations:** 1 Karl Donath Laboratory for Hard Tissue and Biomaterial Research, University Clinic of Dentistry, Medical University of Vienna, 1090 Vienna, Austria; 2 Austrian Cluster for Tissue Regeneration, 1200 Vienna, Austria; 3 Division of Oral Surgery, University Clinic of Dentistry, Medical University of Vienna, 1090 Vienna, Austria; 4 Ludwig Boltzmann Institute for Traumatology, The Research Center in Cooperation with AUVA, 1200 Vienna, Austria

**Keywords:** sinus floor augmentation, graft packing density, macro-porosity, bone substitutes, tissue distribution, interparticle spacing, human, bone regeneration, histomorphometry, gradient

## Abstract

**Simple Summary:**

Following tooth loss, the jaw bone undergoes gradual atrophy of the tooth-bearing alveolar process which poses a major challenge for dental implant therapy. In order to compensate for the decreasing bone height and bone volume in the upper jaw, bone graft particles can be inserted into the maxillary sinus (maxillary sinus floor augmentation). The native bone ideally integrates these particles, proving an increased bone supply for subsequent implant placement. Despite the longstanding clinical application of this surgical procedure, there is still no scientific rationale for whether particulate bone grafts should be compressed or lightly packed. We therefore evaluated the spatial distribution of bone substitute particles in human maxillary sinus biopsies and investigated the association between bone graft packing and bone regeneration 6 months after maxillary sinus floor augmentation. In fact, bone graft particles were not homogeneously distributed over the length of biopsies. With increasing distance from the native bone of the sinus floor, the number of predominantly small, densely packed bone graft particles increased, which appeared to be detrimental to graft integration. These findings suggest that excessive compaction of bone graft particles should be avoided in order to optimise the macrostructural environment for bone regeneration in maxillary sinus floor augmentations.

**Abstract:**

Research in maxillary sinus floor augmentation (MSFA) focussed on the optimisation of microstructural parameters such as microporosity and particle size of bone substitute particles (BS). However, little is known about the impact of BS packing and the corresponding (void) interparticular space on bone regeneration. The aim of this study was to characterise the spatial distribution of BS and its association with BS integration 6 ± 1 months after MSFA. Histological thin-ground sections of 70 human sinus biopsies were histomorphometrically analysed: In serial zones of 100 µm proceeding from the sinus floor (SF) up to the apical end of the biopsy, we measured the distribution of BS particles within these zones in terms of volume (BSV/TV), number and size of BS particles, interparticle spacing (BS.Sp) and bone-to-BS contact. BS particles were not homogeneously distributed over the length of biopsies: The first 200 µm directly adjacent to the SF represented a zone poor in BS particles but with high osteogenic potential. Graft packing density increased from the SF towards the apical part of the AA. Integration of BS particles was inversely associated with the distance to the SF and the graft packing density. A high packing density through excessive compaction of BS particles should be avoided to optimise the macrostructural environment for bone regeneration.

## 1. Introduction

Bone substitute materials are medical products with osteoconductive and hydrophilic properties that find a widespread application in orthopaedics, dentistry, and maxillofacial surgery. Despite the vast number of publications dealing with bone augmentation treatments, little is known about the principal mechanisms that lead to therapeutic success. Maxillary sinus floor augmentation (MSFA) is a predictable augmentation method to increase alveolar bone height in severely atrophied posterior maxillae prior to implant placement [1,2]. In this surgical procedure, bone substitute (BS) particles are inserted into the space created by the elevation of the Schneiderian membrane (SM). Ideally, bone is stimulated to grow into the augmented area (AA), thereby integrating the BS particles and increasing bone supply and stability for dental implants [3,4,5]. The regeneration potential of the local native bone of the sinus floor (SF) and the sinus walls thus plays a decisive role in successful MSFA [6,7,8].

Various particulate bone grafts from different sources are applied in clinical practice nowadays. These grafting materials are characterised by their osteoinductive and osteoconductive properties. While autografts comprise numerous growth factors and autologous cells that can directly exert their osteoinductive potential in the host tissue, xenografts (e.g., deproteinised bovine bone mineral, DBBM) only passively contribute to the bone regeneration process: they provide an osteoconductive surface that allows signalling proteins to adhere, cells of the host tissue to attach, proliferate, differentiate and deposit new bone (nB) [9].

The size and porosity of the BS particles are considered critical parameters in this context [10]. Smaller BS particles are reported to promote graft resorption and nB formation, whereas larger particles provide greater volume stability and preservation of bone volume within grafted defects [10,11,12]. However, large BS particles take up more space in an AA and offer less surface area in relation to their volume than smaller particles [13]. Porous BS particles are favoured as the pores increase the total surface area for bone formation: a three-dimensional network of micro- and macropores facilitates the transport of nutrients and oxygen, invasion of cells, and thus vascularisation—A prerequisite for nB formation [14]. Pore sizes between 100 and 300 µm are assumed to favour bone formation [14,15].

Considering these structural aspects, it becomes evident that also the three-dimensional packing of BS particles and the distance between the individual particles within AA constitutes a macroporous network. The spatial distribution of BS particles and the available space in-between determine “macroporosity” at a higher structural level [16,17]. Although numerous studies deal with the optimised porosity and particle size of bone grafts [15], little is known about the impact of their packing density on the therapeutic success in MSFA [17,18]. Like in socket/ridge preservation [19], there is still no biological or scientific rationale for whether particulate bone grafts should be compressed or lightly packed. Therefore, quantitative data on the actual three-dimensional packing condition found in the augmented sinus is vitally needed to derive evidence-based recommendations for clinical practice. Especially patients with compromised bone healing might benefit from an optimised macrostructural environment in the augmented space.

In our latest study, we reported that nBF follows a gradient from the native bone of the SF towards the apical region of the AA [20]. This current study seeks to contribute to the understanding of the mechanisms of bone regeneration depending on the compaction of bone graft particles.

Therefore, the aim of this basic research was to characterise the vertical distribution, number, size, and interparticle space of BS particles in human sinus biopsies six months after MSFA. We hypothesised that the distribution of BS follows an inverse gradient to nBF in the biopsy and therefore quantified the spatial distribution of tissues in serial zones of 100 µm continuously over the full length of biopsies. Clinical observation suggests that, particularly in the apical region of AA, BS particles often remain separated and are not integrated into newly formed bone [20,21]. Based on this, we analysed if the incorporation/consolidation of BS particles into new bone is related to graft packing density and the distance to native bone from the sinus floor.

## 2. Material & Methods

This study comprises sinus biopsies of MSFA provided by seven Medical University Clinics from Austria (n = 2) and Germany (n = 5) [22,23,24]. All biopsies were processed into histological thin ground sections in the Karl Donath Laboratory for Hard Tissue and Biomaterial Research, University Clinic of Dentistry, Medical University of Vienna, Austria. Based on these samples, two multicentre studies have been conducted investigating the effect of the maxillary region and patients’ age and sex on bone regeneration after MSFA [25] and the vertical gradient of bone regeneration [20]. The present study investigates parts of the histological material from a novel scientific perspective that has not been studied to date, viz the characterisation of the bone graft packing and its association with bone regeneration.

This study was conducted according to the guidelines of the Declaration of Helsinki and approved by the local Ethics committee boards of Austria and Germany [Austria: 102/2004, 22/2007, 18-053 ex 06/07; Germany: 837.274.04 (4432)].

### 2.1. Study Sample

Patients aged >18 years, with a residual alveolar bone height of <5 mm, requiring at least one dental implant in the premolar or molar region of the posterior maxilla, underwent a two-stage approach of MSFA. Patients were not included in cases of tooth extraction at the implantation site, periodontal disease, pathological conditions of the maxillary sinus, metabolic or degenerative diseases of bone (e.g., diabetes mellitus, hyperparathyroidism, osteoporosis), long-term medication with NSAID or corticosteroids, cigarette smoking (>5 cigarettes/day), alcoholism, and complications due to MSFA.

### 2.2. MSFA and Sinus Biopsies

Patients were subjected to MSFA procedures via the lateral window technique [3,4]. After a healing period of 6 ± 1 months, biopsies (Ø 2–3.2 mm) were taken using a trephine burr, and implants were installed in the drill holes.

Two inclusion criteria for biopsies were applied:

(1) MSFA with deproteinised bovine bone mineral (DBBM; BioOss, Geistlich Pharma, Wolhusen, Switzerland) alone or in combination with adjuncts (autologous bone (aB) harvested intraorally, culture-expanded aB cells isolated from the anterior iliac crest, aB with platelet concentrate, aB with mesenchymal stem cells aspirated from the tibia). The potential confounding effect of various adjuncts was statistically considered.
(2) Presence of both the native bone of the SF and the augmented area.

In total, 70 biopsies of 46 patients (30 ♀, 16 ♂) met the inclusion criteria and provided the study sample.

### 2.3. Histology

Biopsies were fixed in a buffered 4% formaldehyde solution, dehydrated in ascending grades of alcohol, and embedded in a light-curing resin (Technovit 7200 VLC + BPO; Kulzer, Wehrheim, Germany). Undecalcified thin-ground sections were produced along the long axis of the biopsies, according to Donath (1988) [26].

41.4% (n = 29) of the sections were stained with Levai-Laczko dye [27] and digitised with a camera mounted on a microscope (Nikon DXM1200/Microphot-FXA, Tokyo, Japan). Multiple single images per specimen were merged into high-resolution overview images (2.212 µm per pixel) (Lucia G 4.71, LIM., Praha, Czech Republic). The remaining 58.6% of the sections (n = 41) were scanned using an SEM (JSM-6310, Jeol, Tokyo, Japan) with back-scattered electrons at 15/20 kV and a resolution of 2.695 µm per pixel. The comparability of the results of the SEM and the histological image sources had previously been verified by the intraclass correlation coefficient within 10 biopsy specimens (≥0.92) [25].

### 2.4. Histomorphometric Analysis

Digital images were semi-automatically segmented, and tissue types were classified: (1) pre-existing/native bone of the SF, (2) newly formed bone, (3) bone substitute material, and (4) soft tissue/marrow area within the augmented area (AA). Particles smaller than 0.008 mm² were considered to be debris and ascribed to void space. Inaccurately classified areas were manually corrected under microscopic control (Adobe Photoshop, Adobe, San Jose, CA, USA). Segmentation and histomorphometric measurements were performed using Definiens Developer XD 2.7 (Definiens, Munich, Germany).

The following histomorphometric parameters were assessed in serial 100 µm zones starting from the native bone of the SF towards the apical end of the biopsies [20].

#### 2.4.1. Bone Substitute Volume Fraction (BSV/TV in %)

**Figure 1:**
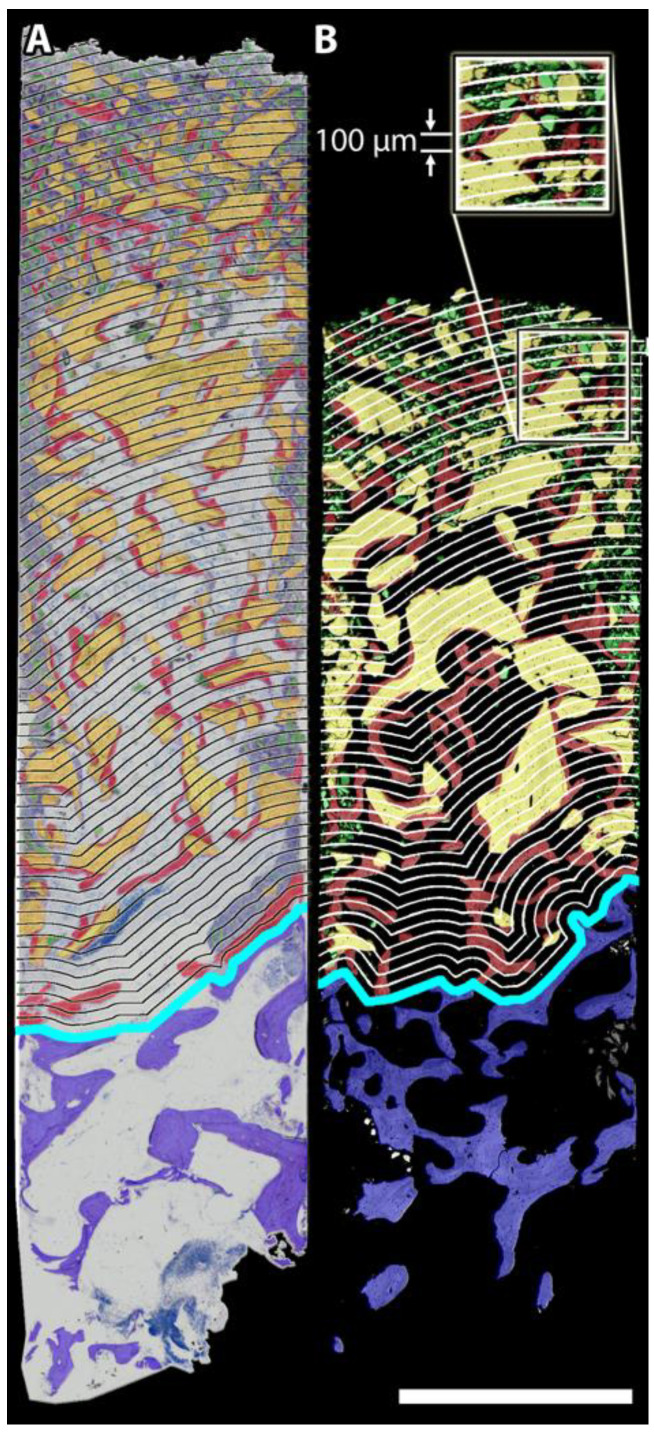
Histological thin-ground section (**A**) and a SEM image (**B**) superimposed with colour-coded classification images to illustrate the vertical distribution of BS particles within the augmented area. Blue: native bone of the sinus floor, red: newly formed bone, yellow: bone substitute particles, green: debris, in-between: marrow area/soft tissue; turquoise: borderline between the sinus floor (SF) and the augmented area. In parallel to the turquoise line, contour lines were set at an interval of 100 μm to measure the histomorphometric parameters within each of the resulting serial zones: bone substitute volume fraction (BSV/TV), number of BS particles (BS.N), average size of BS particles (avgBSV), interparticle spacing (BS.Sp), bone-to-bone substitute contact (BBSC in %) and bone formation in terms of bone volume absolute (BV), bone volume per available volume (BV/Av.V), composite volume fraction (Co.V/TV). The distribution over the entire length of the augmented area (AA) was calculated for all biopsies. The results support our hypothesis that BS particles were not uniformly distributed over the length of the AA six months after MSFA but typically followed specific patterns: In close proximity to the SF (turquoise line), packing density was lowest, characterised by low BSV/TV and large interparticle spacing (BS.Sp). BS particles tended to be well integrated into nB. With increasing distance to the SF (>2 mm), BSV/TV was higher, and particles were more densely packed, showing reduced “osseointegration”. Interestingly, the most proximal 200 µm adjacent to the SF represent a zone relatively poor in BS particles in the majority of biopsies. Scale bar 2 mm.

The native bone of the SF was separated from the augmented area with a manually drawn line. In parallel to this “sinus floor borderline”, contour lines were set at an interval of 100 µm over the entire length of the augmented area. The volume of the BS (BSV) was measured within each of the created serial 100 µm zones (i.e., tissue volume; TV), beginning from the zone adjacent to the SF up to the apical top of the augmented area (Figure 1) [20]. Based on these values, the gradient of BSV/TV was calculated.

#### 2.4.2. Number of BS Particles (BS.N)

While the gradient of BSV/TV describes the spatial distribution of total BS volume within the augmented area, it provides no information about the number and size of the individual BS particles.

A measure analogous to the trabecular number was used to quantify the distribution of the number of BS particles. The number of BS particles (BS.N) was defined as the inverse of the mean spacing of the mid-axes of BS particles. This measurement can only be calculated in the areas between particles. To show how the individual BS particles were distributed over the entire length of the AAs, the mean number of BS particles per zone was assessed and plotted against the distance from the SF.

#### 2.4.3. Average Size of BS Particles (avgBSV in mm²)

In addition to BS.N, the size of individual BS particles also affects the spatial organisation within the AA. Accordingly, a rule set was created to assign a unique number to each BS particle, starting with the particle located closest to the “sinus floor borderline” (i.e., in zone 0–100 µm in the augmentation area) towards the most apical particles in the AA (Definiens Developer XD, Definiens, Munich, Germany). If neighbouring BS particles were not automatically identified as single particles in the segmentation process, they were separated with a manually drawn line using Adobe Photoshop (Adobe, San Jose, CA, USA). Since most BS particles occupy more than one 100 µm zone, the numbered particles were assigned to the 100 µm zone where their respective centre of mass lies in.

Based on this assignment, the volume of each individual particle was calculated, and their average volume per 100 µm zone was plotted as a gradient of avgBSV over the length of the AA.

#### 2.4.4. Interparticle Spacing (Mean Distance between BS Particles; BS.Sp in mm)

Interparticle spacing refers to the mean distance between individual BS particles (comparable to “trabecular separation”/Tb.Sp measurement) as a measure of BS packing density.

Since bone regeneration within the AA requires not only a surface area for bone cells to attach but also sufficient space to grow into, the mean distance between single BS particles was assessed in the next step, representing BS separation (BS.Sp). When BS particles are tightly packed, then the intra-particular space and thus BS.Sp is low. High intra-particular space and thus high BS.Sp indicates a low packing density of BS particles within the respective zones.

Interparticle spacing was measured by assigning each pixel between BS particles the diameter of the largest circle which fits between the particles and encompasses the respective pixel. BS.Sp is the average of these values over the whole area between particles.

#### 2.4.5. Bone-to-Bone Substitute Contact (BBSC in %)

To quantify how well BS particles were integrated into newly formed bone (nB) over the length of the AA, BBSC was calculated for all serial 100 µm zones as the percentage of the BS surface length that is in direct contact with nB.

#### 2.4.6. New Bone Formation in Terms of Bone Volume Absolute (BV in mm²) and Bone Volume Per Available Volume (BV/Av.V in %)

BV measures new bone formation in the AA in absolute numbers, whereas BV/Av.V quantifies the fraction of newly formed bone within the available volume, viz., the void volume not occupied by BS particles.

#### 2.4.7. Composite Volume Fraction (Co.V/TV in %)

As a measure of the total amount of potentially biomechanically active solid material within the AA [28,29], the composite volume consisting of newly formed BV plus BS volume per tissue volume was calculated.

### 2.5. Statistics

Mean and standard deviation (SD) were calculated for all parameters over the entire AA and at several distances from the SF (0.1 mm, 1.5 mm, 3 mm, 6 mm and 9 mm) in windows of 0.5 mm width. Scatter plots for all parameters were plotted, including local polynomial regression lines to describe the general shape of the data.

For the primary measurements BSV/TV and BBSC, we estimated inductive linear mixed models [30], which included the biopsy ID as random factors. DBBM and related groupings were included as random factors initially but removed from the final models due to very low variance estimates. To model the shape of the curves, we included both the distance from the SF, as well as the natural logarithm of the distance from the SF as fixed effects. Tests were calculated using Satterthwaite′s method [31]. All computations were done using R version 4.1.2 [32].

## 3. Results

In total, 70 biopsies of 46 patients (30 ♀, 16 ♂) with a mean age of 53.3 years (SD 9.8) were evaluated (1–3 biopsies per patient); Of these, 30 biopsies were harvested from the premolar, and 40 from the molar region. The mean length of the augmented area of the biopsies was 5.2 mm (SD: 2.5). BSV/TV and BBSC were primary measurements; all other parameters were secondary measurements.

The variation of measured parameters was relatively high, reflecting the biological variability found in clinical practice. The potential confounding effect of various adjuncts was statistically considered. Several marked patterns in the spatial distribution were identified.

### 3.1. Spatial Distribution of BSV/TV

**Figure 2:**
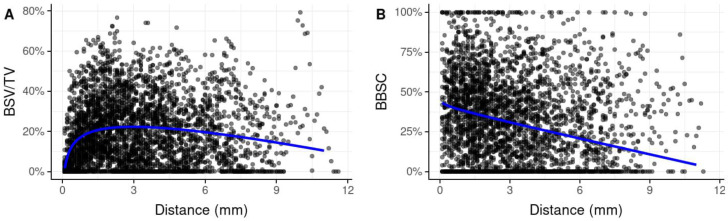
Scatter plots including a regression line illustrating the vertical distribution of the primary parameters BSV/TV and BBSC over serial zones of 100 µm across the entire length of biopsies starting from the native bone of the SF (0 mm zero reference) towards the apical end of the biopsies. (**A**) BSV/TV: In immediate proximity to the SF, expected BSV/TV was very low. At a distance of approximately 3 mm from the SF, it reached a maximum with 22.9% before it steadily decreased with increasing distance from the SF. Both, the logarithmic increase of BSV/TV (most evident within the first 1.5 mm) and the linear decrease were statistically significant (*p* < 0.001). (**B**) BBSC: The few, mostly large particles that were typically located in the area close-by the SF (0–1.5 mm) were well integrated into newly formed bone. At a distance of approximately 0.1 mm from the SF, 41.0% of the BS surface was in contact with new bone. With increasing distance, BBSC decreased steadily. The linear decrease of BBSC with distance from the SF was statistically significant. (*p* < 0.001). BSV/TV: bone substitute volume per tissue volume; BBSC: bone-to-bone substitute contact; SF: sinus floor.

Mean BSV/TV within the entire AA was 19.2% (SD 16.4) (Table 1). A closer look at the spatial distribution of BSV/TV within the AA reveals a marked increase of BSV/TV within the first 1.5 mm adjacent to the SF and a subsequent steady decline (Figure 2A). In immediate proximity to the SF (within a distance of 0.1 mm to the SF) expected BSV/TV was very low (1.7%). At a distance of approximately 3 mm from the SF, it reached a maximum of 22.3% before it steadily decreased to 19.6% at 6 mm and 14.5% at 9 mm (Table 2). Both the logarithmic increase of BSV/TV (most evident within the first 1.5 mm) and the linear decrease were statistically significant (*p* < 0.001).

### 3.2. Distribution of the Number of BS Particles (BS.N)

On average, 1.4 BS particles (SD: 0.7) are to be expected in an arbitrary 1 mm zone across the entire AA (Table 1). However, BS particles were not homogeneously distributed over the length of the AA. Close to the SF, significantly fewer BS particles tended to be present (0.64 BS particles/mm at a distance of 0.1 mm from the SF). Within the first approx. 1.5 mm of the AA, the number of BS particles increased markedly, reaching a plateau at approx. 2 mm (1.5 particles/mm) (Table 3).

### 3.3. Distribution of the Mean BS Particle Size (avgBSV)

The mean size of individual BS particles within the entire AA was 0.1 mm² (SD: 0.10), ranging from 0.01 mm² to 1.6 mm² (Table 1). The size of the individual BS particles declined with increasing distance from the SF: In immediate proximity to the SF, BS particles tended to be larger than in the more apical zones. In the first 1.5 mm of the AA, an average BS particle had a size of approx. 0.09 mm². At a distance of approx. 6 mm, avgBSV had almost halved to 0.05 mm². The relatively low number of measurements at a distance of >8 mm might be responsible for the slight increase at 9 mm (Table 3).

Overall, with increasing distance from the SF, the number of BS particles tended to increase, while the size of particles decreased steadily.

### 3.4. Interparticle Spacing (BS.Sp)

The average BS interparticle space (i.e., mean distance between BS particles as a measure for BS packing density) in an arbitrary 1 mm zone of a biopsy was 0.65 mm (SD 0.58) (Table 1). In close proximity to the SF, the distance between individual BS particles was highest at 0.90 mm, corresponding to a lower packing density. With increasing distance from the SF, BS.Sp declined and stabilised at a distance of >1.5 mm from the SF reaching approx. 0.64 mm, which corresponds to a denser BS packing in this region. (Table 3). Variation, however, was very high in all zones of the biopsies.

### 3.5. Bone-to-Bone Substitute Contact (BBSC)

Mean BBSC within the entire AA was 33.5% (SD 26.6) (Table 1). While BSV/TV was lowest in the region adjacent to the SF, BBSC was highest in this region. The few, mostly large particles that were typically located in this area close-by the SF (0–1.5 mm), were well integrated into newly formed bone. At a distance of 0.1 mm from the SF, 43.2% of the BS surface was in contact with nB, at approx. 1.5 mm from the SF BBSC was 36.4%. With increasing distance, BBSC decreased steadily to 31.0% at a distance of 3 mm, 20.8% at 6 mm and 10.8% at 9 mm. The linear decrease of BBSC with distance from the SF was statistically significant. (*p* < 0.001) (Table 2, Figure 2).

### 3.6. New Bone Formation (BV, BV/AV)

By absolute numbers, the volume of newly formed bone was highest in the area closest to the SF. In intimate proximity to the SF, 0.09 mm² nB was formed. Within the first 3 mm, BV decreased significantly and levelled off at approx. 0.05 mm² per zone (Table 3).

A closer look at the BV per available volume, however, reveals that the percentage of nB formation within the unoccupied, void space within the AA is relatively constant, in the range of approximately 25%. When much space is available (i.e., close to SF where BSV fills less than 5% of TV), this void volume was filled with nB up to approx. 25% BV/AV. Similarly, when a large space is already occupied by BS particles (i.e., >3 mm from the SF where BSV fills approx. 20% of TV), nB fills up the remaining void space also to a level of approx. 23–24% (Table 3).

### 3.7. Composite Volume (Co.V/TV)

The composite volume of mineralised material (BSV plus BV per TV) that can be regarded as potentially “biomechanically active” was 38.8% (SD 18.3) within the entire AA (Table 1). Due to the low BSV/TV adjacent to the SF, Co.V/TV was lowest in this region with 28%, reaching a peak at approx. 1.5 mm with 45%, and slightly levelled off to 38% at approx. 6 mm (Table 3).

### 3.8. Correlation between BBSC and BSV/TV

**Figure 3:**
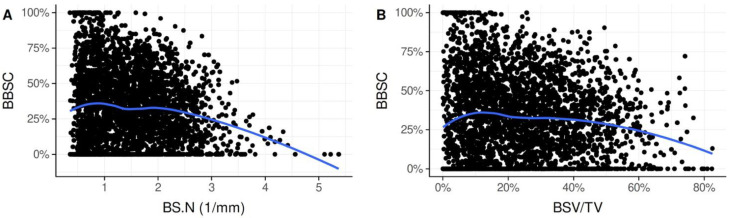
Scatter plots showing the association between BS.N and BBSC (**A**) and between BSV/TV and BBSC (**B**). Integration of BS is highest when 5–20% of the volume is occupied by BS particles. A high volume of BS per TV (>60%) and a high number of particles (>2.5 particles/zone) are associated with reduced osseointegration. BBSC: bone-to-bone substitute contact; BS.N: bone substitute number; BSV/TV: bone substitute volume per tissue volume.

Despite the high variation in BSV/TV and BBSC, there was an overall-trend showing an association between these two parameters. When BSV/TV was about 5–20%, BBSC reached a maximum of approx. 36%. The more BS particles present in a zone (>60%), the smaller the percentage of BS surface that was in direct contact with nB (BBSC < 25%), meaning that particles were less integrated into nB (Figure 3B).

### 3.9. Correlation between BBSC and BS.N

Furthermore, the number of BS particles was associated with BBSC. When BS.N was low with 1–2 particles per mm, BBSC reached a maximum of approx. 36%. The more BS particles present (>2.5 particles/zone), the smaller the BBSC (<25%) (Figure 3A).

## 4. Discussion

In this study, we analysed bone graft packing in 70 human maxillary sinus biopsies and its association with bone regeneration. We thereby seek to contribute to the understanding of bone regeneration mechanisms depending on the compaction of bone graft particles.

Traditionally, histomorphometric parameters characterising bone graft and bone regeneration within the augmented sinus are calculated as mean values for the entire volume of a biopsy. However, this kind of evaluation conceals any information about the spatial distribution of tissues that might be of clinical relevance. Quantitative data on the actual three-dimensional packing conditions found in the augmented sinus is needed to provide a scientific rationale for whether particulate bone grafts should be compressed or lightly packed.

In the present study, total BSV/TV amounted to approx. 20%, which is in accordance with others [33,34,35,36,37]. A closer look at the serial 100 µm zones, however, revealed that BS particles were not homogeneously distributed over the length of the AA but followed specific patterns:

(1) Graft packing density was significantly lower in proximity to the sinus floor (<1.5 mm) than in the more apical area. Especially, the first 200 µm directly adjacent to the SF represent a zone poor in BS particles but rich in new bone formation.
(2) Integration of BS particles into newly formed bone was associated with the distance from the SF and graft packing density. In concordance with histological observations [20,21], apical particles were typically more densely packed and less integrated into nB.

The observed graft packing gradient is in line with other studies that analysed BSV in a limited number of discrete zones in grafted sinus biopsies [7,36,38,39]. Pignaton et al. (2020) examined the most proximal 4 mm in 101 biopsies (21 patients) grafted with anorganic bovine bone in four discrete zones of 1 mm [36]. The authors reported that the BS area was lowest in the 1st mm zone (adjacent to native bone) with 18.94%, increased to a maximum of 23.33% in the 2nd mm zone, and levelled off to 19.67% in the 4th mm zone. By analysing BSV in serial zones of 100 µm, we were able to specify the distribution of BS particles within the first mm in greater detail. Interestingly, there was a significant increase of BSV within the most proximal mm from 1.6% at 0.1 mm to 14% at 0.5 mm and 19% at 1.0 mm from the SF that has not been identified in previous studies so far.

Within the augmented maxillary sinus, the individual BS particles form a three-dimensional macrostructural, “porous” network [40]. Physically speaking, porosity is defined as the percentage of void space in a volume of a solid object. It depends on several parameters, such as packing density and particle size distribution [41,42]. The size and shape of BS particles, as well as the manual compaction of the graft, thus have a decisive impact on interparticle spacing and packing density in MSFA. To characterize this “macro-porosity” of the BS network in AA as a whole, not only the volume, but also the number and size of BS particles (referring to the solid object) and the distance between individual BS particles (referring to the void inter-space) were histomorphometrically analysed over the entire length of the biopsies.

The mean distance between the BS particles (BS.Sp) was 0.6 mm, which meets the reported criterion of “macroporosity” favouring osteogenesis [14]. Martinez et al. (2011) also measured the distance between single ABB particles along the lines of an overlaid grid and reported a lower mean interparticular distance of 0.34 mm 8 months after MSFA [16]. The distinct measurement methods might be responsible for the diverging results.

A closer look at the spatial distribution of BS particles in our study revealed that the interparticular distance (BS.Sp) decreased significantly with the distance from the SF, the number of individual particles, however, increased. In fact, in 81.5% of the biopsies, fewer BS particles were present in the 1st mm adjacent to the SF than in the next 3 mm towards the Schneiderian membrane. In other words, BS packing density increased towards the Schneiderian membrane.

Our findings demonstrate that the first 200 µm directly adjacent to the SF represent a zone poor in BS particles. The reason for this phenomenon is not clear. From a clinical point of view, it may be speculated that the low packing density adjacent to the SF might be the consequence of micro-movements within the maxillary sinus after MSFA. Since the Schneiderian membrane constantly moves and stretches due to the airflow during the breathing of the patient, it is conceivable that the particles become aggregated and compacted against themselves toward a core in the centre of the sinus which would explain the lower density of BS particles adjacent to the SF.

Interestingly, this relatively “BS-deficient zone” adjacent to the SF is particularly rich in newly formed bone. From a biological point of view, it is plausible that the elevation of the Schneiderian membrane from native bone might play a critical role in this context [43]. In this procedure, the Schneiderian membrane is detached from the native bone of the SF (and lateral walls), which leads to the rupture of blood vessels, formation of blood clots and the stimulation of the periosteum [44,45,46]. Since the inner cambium layer of the periosteum possesses a high osteogenic potential, it is assumed that the adjacent zone is particularly active [47,48]. The inserted, not yet stabilised BS particles might exert additional stress on the exposed native bone due to minimal particle movements. As the osteogenic field proceeds from the native bone forward into the AA, BS particles are assumed to migrate apically. With increasing nB formation, the space for movement becomes continuously reduced, and marginal BS particles are integrated until the augmentation is stabilised. The “BS-deficient” zone adjacent to the SF might arise from this initial phase of bone formation until stable conditions and primary anchorage of BS particles are achieved.

This present study demonstrates that the presence of BS particles appears not to be mandatory for nB formation adjacent to the SF, which is supported by the fact that nB is also formed in membrane elevation without BS particles [49]. In implant healing, this basal periosteal zone which implies the gusset between the host bone and the most basal implant thread, is also reported as highly osteogenic [17,45]. Further research is needed to elucidate the mechanism underlying the observed gradient of BSV within the AA. It remains to be investigated if biomechanical differences (with regard to implant stability) exist between this “BS-deficient” zone and the adjacent “composite zone”, where BS is integrated into nB.

Another important finding of this study is that “osseointegration” of BS particles appears to be inversely related to the quantity of BS. In fact, bone regeneration and BS integration not only requires a surface area for bone cells to attach but also sufficient (interparticular) space for bone to grow into [50]. Since BSV was not homogeneously distributed over the length of the AA, the available “void” space for bone in-growth varied significantly. As expected, the absolute volume of nB formation was highest adjacent to the SF and declined with increasing distance (BV: 0.09 mm² at 0.1 mm to 0.04 mm² at 9 mm) which is in line with other studies [6,7,51,52]. The same gradient was observed for the integration of BS particles into nB (BBSC: 41% at 0.1 mm to 8.6% at 9 mm). Particularly small particles in the apical area of the AA tend to be poorly integrated into nB [20,21]. A high BS packing density characterised by a high volume of BS particles and small interparticular spacing appeared to have an unfavourable effect on BS integration. A low BS volume, by contrast, did not seem to impede bone regeneration in this setting. This is in accordance with histological observations and a recent systematic review by Pesce et al. reporting that the presence of a larger volume of BS particles did not necessarily results in a larger volume of nB formation [53]. These findings suggest that a high graft packing density through excessive compaction of BS particles should be avoided in order to optimise the macrostructural environment for bone regeneration after MSFA.

By contrast, Romanos et al. (2018) found increased bone regeneration in small calvaria defects in the rabbit when a compressive force of 8.2 g was applied to particulate bone graft material [18]. Delgado-Ruiz et al. (2015) reported increased new bone formation in extraction sockets of dogs with a higher compressive force (200 g) compared to a lower force (50 g or 10 g) [19]. It was discussed that the graft particles transmit load to the bony walls of a defect which might accelerate bone response and thus bone formation. Curiously, however, most new bone formation was found in the ungrafted control group, which supports our findings that the overfilling and high compression of particulate bone grafts does not stringently lead to superior bone formation. Since the biomechanical situation and the dimensions of the augmented sinus differ considerably from the rather narrow extraction socket or calvaria defect, a reliable comparison of these results is precluded.

Another interesting aspect of this study is that the portion of new bone within the available volume seemed relatively constant with approx. 25%. Regardless of how much volume was already occupied by BS particles, nB filled up the remaining void space to a level of approx. 25%. It remains to be elucidated if this result might represent some kind of biological threshold in the augmented maxillary sinus. The composite volume comprising new BV plus BSV per TV as an indicator for the potentially biomechanically active solid material revealed a different picture. Composite volume amounted to 26% close to the SF, 45% at 1.5 mm and then levelled off to approx. 38% in farther distance to SF, which is in line with others [37,39]. In fact, composite volume is within the range of maxillary posterior trabecular bone (approx. 38%) [54] and also meets the minimum requirements of vital bone to sustain osseointegration of implants [55].

The major limitation of this study is the heterogeneity of the sample. However, statistical methods did not show any evidence of a potential confounding effect of DBBM and its combinations with adjuncts (aB/cells) on the hypotheses under study.

Since we only analysed biopsies harvested 6 months after MSFA, the results capture a “snapshot” of the packing condition and no information about temporal dynamics of spatial nB formation can be provided. Moreover, biopsies only depict conditions of the implantation site and might not be representative for the entire augmented sinus.

Further studies are needed to evaluate the impact of different standardised packing densities on bone regeneration in MSFA to provide a basis for evidence-based recommendations in clinical practice.

## 5. Conclusions

Six months after maxillary sinus floor augmentation, bone substitute (BS) particles were not homogeneously distributed over the length of the augmented area. The first 200 µm directly adjacent to the sinus floor represents a zone poor in BS particles but with high osteogenic potential. With increasing distance from the SF, the number of predominantly small, densely packed BS particles increases, which appears to be detrimental to graft consolidation.

These findings suggest that a high graft packing density through excessive compaction of BS particles should be avoided in order to optimise the macrostructural environment for bone regeneration in maxillary sinus floor augmentations.

## Figures and Tables

**Table 1:** Descriptive statistics for histomorphometric parameters measured over the entire length of the biopsies. Values are presented as mean ± standard deviation (SD).

	Measurement Unit	Mean	SD
**BSV/TV**	(%)	19.2	16.4
**BS.N**	(1/mm)	1.4	0.7
**avgBSV**	(mm²)	0.1	0.1
**BS.Sp**	(mm)	0.6	0.6
**BBSC**	(%)	33.5	26.6
**BV**	(mm²)	0.1	0.0
**BV/AV**	(%)	24.2	16.2
**Co.V/TV**	(%)	38.8	18.3

**Abbreviations:** BSV/TV, bone substitute volume per tissue volume; BS.N, bone substitute number; avgBSV, average BS volume; BS.Sp, interparticle spacing; BBSC, bone-to-bone substitute contact; BV, bone volume absolute; BV/AV, bone volume per available volume; Co.V/TV, composite volume (=BSV plus BV) per tissue volume; SD, standard deviation.

**Table 2:** Regression predictions for the primary measurements BSV/TV and BBSC in 0.5 mm windows at certain distances from the SF.

Distance from SF	BSV/TV	BBSC
(mm)	(%)	(%)
**0.1**	1.7	43.2
**1.5**	20.7	36.4
**3.0**	22.3	31.0
**6.0**	19.6	20.8
**9.0**	14.5	10.8

**Abbreviations:** SF, sinus floor; BSV/TV, bone substitute volume per tissue volume; BBSC, bone-to-bone substitute contact.

**Table 3:** Descriptive statistics for histomorphometric parameters (secondary measurements) in 0.5 mm windows at certain distances from the sinus floor: In order to illustrate the spatial distribution of the histomorphometric parameters at certain distances from the SF, measurements were averaged in 0.5 mm windows starting from the borderline of the native bone of the SF (0 mm representing the zero reference) towards the apical end of biopsies. Values are presented as mean ± standard deviation.

Distance from SF	BS.N	avgBSV	BS.Sp	BV	BV/AV	Co.V/TV
(mm)	(BS Particles/mm)	(mm²)	(mm)	(mm²)	(%)	(%)
**0.1**	0.64 ± 0.27	NaN ± NA	0.90 ± 0.81	0.09 ± 0.08	25.5 ± 18.2	28.1 ± 18.2
**1.5**	1.51 ± 0.64	0.09 ± 0.14	0.64 ± 0.46	0.06 ± 0.04	27.5 ± 15.3	45.1± 16.2
**3**	1.39 ± 0.74	0.07 ± 0.09	0.64 ± 0.60	0.05 ± 0.04	23.7 ± 17.1	37.9 ± 19.7
**6**	1.52 ± 0.72	0.05 ± 0.05	0.61 ± 0.50	0.05 ± 0.03	22.7 ± 12.2	39.2 ± 14.5
**9**	1.90 ± 0.60	0.07 ± 0.07	0.38 ± 0.35	0.04 ± 0.03	32.8 ± 20.8	43.8 ± 20.3

**Abbreviations**: SF, sinus floor; BSV/TV, bone substitute volume per tissue volume; BS.N, bone substitute number; avgBSV, average BS volume; BS.Sp, interparticle spacing; BBSC, bone-to-bone substitute contact; BV, bone volume absolute; BV/AV, bone volume per available volume; Co.V/TV, composite volume (=BSV plus BV) per tissue volume.

## Data Availability

Restrictions apply to the availability of these data. Data are available from the authors with the permission of University Clinics providing the histological specimens.

## References

[1] Starch-Jensen T., Jensen J.D. (2017). Maxillary Sinus Floor Augmentation: A Review of Selected Treatment Modalities. J. Oral Maxillofac. Res..

[2] Raghoebar G.M., Onclin P., Boven G.C., Vissink A., Meijer H.J.A. (2019). Long-Term Effectiveness of Maxillary Sinus Floor Augmentation: A Systematic Review and Meta-Analysis. J. Clin. Periodontol..

[3] Tatum H. (1986). Maxillary and Sinus Implant Reconstructions. Dent. Clin. N. Am..

[4] Boyne P.J., James R.A. (1980). Grafting of the Maxillary Sinus Floor with Autogenous Marrow and Bone. J. Oral Surg..

[5] Park W.-B., Kang K.L., Han J.-Y. (2019). Factors Influencing Long-Term Survival Rates of Implants Placed Simultaneously with Lateral Maxillary Sinus Floor Augmentation: A 6- to 20-Year Retrospective Study. Clin. Oral Implant. Res..

[6] Avila G., Wang H.-L., Galindo-Moreno P., Misch C.E., Bagramian R.A., Rudek I., Benavides E., Moreno-Riestra I., Braun T., Neiva R. (2010). The Influence of the Bucco-Palatal Distance on Sinus Augmentation Outcomes. J. Periodontol..

[7] Kolerman R., Nissan J., Rahmanov M., Calvo-Guirado J.L., Green N.T., Tal H. (2019). Sinus Augmentation Analysis of the Gradient of Graft Consolidation: A Split-Mouth Histomorphometric Study. Clin. Oral Investig..

[8] Stacchi C., Rapani A., Lombardi T., Bernardello F., Nicolin V., Berton F. (2022). Does New Bone Formation Vary in Different Sites within the Same Maxillary Sinus after Lateral Augmentation? A Prospective Histomorphometric Study. Clin. Oral Implant. Res..

[9] Bauer T.W., Muschler G.F. (2000). Bone Graft Materials: An Overview of the Basic Science. Clin. Orthop. Relat. Res..

[10] Yamada M., Egusa H. (2018). Current Bone Substitutes for Implant Dentistry. J. Prosthodont. Res..

[11] Pallesen L., Schou S., Aaboe M., Hjørting-Hansen E., Nattestad A., Melsen F. (2002). Influence of Particle Size of Autogenous Bone Grafts on the Early Stages of Bone Regeneration: A Histologic and Stereologic Study in Rabbit Calvarium. Int. J. Oral Maxillofac. Implant..

[12] Testori T., Wallace S.S., Trisi P., Capelli M., Zuffetti F., Del Fabbro M. (2013). Effect of Xenograft (ABBM) Particle Size on Vital Bone Formation Following Maxillary Sinus Augmentation: A Multicenter, Randomized, Controlled, Clinical Histomorphometric Trial. Int. J. Periodontics Restor. Dent..

[13] Leiblein M., Koch E., Winkenbach A., Schaible A., Nau C., Büchner H., Schröder K., Marzi I., Henrich D. (2020). Size Matters: Effect of Granule Size of the Bone Graft Substitute (Herafill^®^) on Bone Healing Using Masquelet’s Induced Membrane in a Critical Size Defect Model in the Rat’s Femur. J. Biomed. Mater. Res..

[14] Karageorgiou V., Kaplan D. (2005). Porosity of 3D Biomaterial Scaffolds and Osteogenesis. Biomaterials.

[15] Henkel J., Woodruff M.A., Epari D.R., Steck R., Glatt V., Dickinson I.C., Choong P.F.M., Schuetz M.A., Hutmacher D.W. (2013). Bone Regeneration Based on Tissue Engineering Conceptions—A 21st Century Perspective. Bone Res..

[16] Martinez A., Franco J., Saiz E., Guitian F. (2010). Maxillary Sinus Floor Augmentation on Humans: Packing Simulations and 8 months Histomorphometric Comparative Study of Anorganic Bone Matrix and β-Tricalcium Phosphate Particles as Grafting Materials. Mater. Sci. Eng. C.

[17] Zhou X., Zhang Z., Li S., Bai Y., Xu H. (2011). Osteoconduction of Different Sizes of Anorganic Bone Particles in a Model of Guided Bone Regeneration. Br. J. Oral Maxillofac. Surg..

[18] Romanos G.E., Delgado-Ruiz R.A., Gómez-Moreno G., López-López P.J., de Val J.E.M.S., Calvo-Guirado J.L. (2018). Role of Mechanical Compression on Bone Regeneration around a Particulate Bone Graft Material: An Experimental Study in Rabbit Calvaria. Clin. Oral Implant. Res..

[19] Delgado-Ruiz R., Romanos G.E., Alexandre Gerhke S., Gomez-Moreno G., Maté-Sánchez de Val J.E., Calvo-Guirado J.L. (2018). Biological Effects of Compressive Forces Exerted on Particulate Bone Grafts during Socket Preservation: Animal Study. Clin. Oral Implant. Res..

[20] Beck F., Reich K.M., Lettner S., Heimel P., Tangl S., Redl H., Ulm C. (2021). The Vertical Course of Bone Regeneration in Maxillary Sinus Floor Augmentations: A Histomorphometric Analysis of Human Biopsies. J. Periodontol..

[21] Tadjoedin E.S., De Lange G.L., Bronckers A.L.J.J., Lyaruu D.M., Burger E.H. (2003). Deproteinized Cancellous Bovine Bone (Bio-Oss^®^) as Bone Substitute for Sinus Floor Elevation. J. Clin. Periodontol..

[22] Fuerst G., Strbac G.D., Vasak C., Tangl S., Leber J., Gahleitner A., Gruber R., Watzek G. (2009). Are Culture-Expanded Autogenous Bone Cells a Clinically Reliable Option for Sinus Grafting?. Clin. Oral Implant. Res..

[23] Payer M., Lohberger B., Strunk D., Reich K.M., Acham S., Jakse N. (2014). Effects of Directly Autotransplanted Tibial Bone Marrow Aspirates on Bone Regeneration and Osseointegration of Dental Implants. Clin. Oral Implant. Res..

[24] Wagner W., Wiltfang J., Pistner H., Yildirim M., Ploder B., Chapman M., Schiestl N., Hantak E. (2012). Bone Formation with a Biphasic Calcium Phosphate Combined with Fibrin Sealant in Maxillary Sinus Floor Elevation for Delayed Dental Implant. Clin. Oral Implant. Res..

[25] Reich K.M., Huber C.D., Heimel P., Ulm C., Redl H., Tangl S. (2016). A Quantification of Regenerated Bone Tissue in Human Sinus Biopsies: Influences of Anatomical Region, Age and Sex. Clin. Oral Implant. Res..

[26] Donath K. (1988). Die Trenn-Dünnschliff-Technik Zur Herstellung Histologischer Präparate von Nicht Schneidbaren Geweben Und Materialien. Präparator.

[27] Laczko J., Levai G. (1975). A Simple Differential Staining Method for Semi-Thin Sections of Ossifying Cartilage and Bone Tissues Embedded in Epoxy Resin. Mikroskopie.

[28] Hruschka V., Tangl S., Ryabenkova Y., Heimel P., Barnewitz D., Möbus G., Keibl C., Ferguson J., Quadros P., Miller C. (2017). Comparison of Nanoparticular Hydroxyapatite Pastes of Different Particle Content and Size in a Novel Scapula Defect Model. Sci. Rep..

[29] Pepelassi E., Perrea D., Dontas I., Ulm C., Vrotsos I., Tangl S. (2019). Porous Titanium Granules in Comparison with Autogenous Bone Graft in Femoral Osseous Defects: A Histomorphometric Study of Bone Regeneration and Osseointegration in Rabbits. BioMed Res. Int..

[30] Bates D., Maechler M., Bolker B.M., Walker S.C. (2015). Fitting Linear Mixed-Effects Models Using Lme4. J. Stat. Softw..

[31] Kuznetsova A., Brockhoff P.B., Christensen R.H.B. (2017). LmerTest Package: Tests in Linear Mixed Effects Models. J. Stat. Softw..

[32] R Core Team (2021). R: A Language and Environment for Statistical Computing.

[33] Corbella S., Taschieri S., Weinstein R., Fabbro M.D. (2016). Histomorphometric Outcomes after Lateral Sinus Floor Elevation Procedure: A Systematic Review of the Literature and Meta-Analysis. Clin. Oral Implant. Res..

[34] Lindgren C., Sennerby L., Mordenfeld A., Hallman M. (2009). Clinical Histology of Microimplants Placed in Two Different Biomaterials. Int. J. Oral Maxillofac. Implant..

[35] Lorenz J., Kubesch A., Korzinskas T., Barbeck M., Landes C., Sader R.A., Kirkpatrick C.J., Ghanaati S. (2015). TRAP-Positive Multinucleated Giant Cells Are Foreign Body Giant Cells Rather Than Osteoclasts: Results From a Split-Mouth Study in Humans. J. Oral Implantol..

[36] Pignaton T.B., Spin-Neto R., Ferreira C.E.d.A., Martinelli C.B., de Oliveira G.J.P.L., Marcantonio E. (2020). Remodelling of Sinus Bone Grafts According to the Distance from the Native Bone: A Histomorphometric Analysis. Clin. Oral Implant. Res..

[37] Stacchi C., Lombardi T., Oreglia F., Alberghini Maltoni A., Traini T. (2017). Histologic and Histomorphometric Comparison between Sintered Nanohydroxyapatite and Anorganic Bovine Xenograft in Maxillary Sinus Grafting: A Split-Mouth Randomized Controlled Clinical Trial. BioMed Res. Int..

[38] Artzi Z., Kozlovsky A., Nemcovsky C.E., Weinreb M. (2005). The Amount of Newly Formed Bone in Sinus Grafting Procedures Depends on Tissue Depth as Well as the Type and Residual Amount of the Grafted Material. J. Clin. Periodontol..

[39] Price A.M., Nunn M., Oppenheim F.G., Dyke T.E.V. (2011). De Novo Bone Formation After the Sinus Lift Procedure. J. Periodontol..

[40] Artzi Z., Weinreb M., Carmeli G., Lev-Dor R., Dard M., Nemcovsky C.E. (2008). Histomorphometric Assessment of Bone Formation in Sinus Augmentation Utilizing a Combination of Autogenous and Hydroxyapatite/Biphasic Tricalcium Phosphate Graft Materials: At 6 and 9 Months in Humans. Clin. Oral Implant. Res..

[41] León y León C.A. (1998). New Perspectives in Mercury Porosimetry. Adv. Colloid Interface Sci..

[42] Nimmo J.R. (2013). Porosity and Pore Size Distribution. Reference Module in Earth Systems and Environmental Sciences.

[43] Solar P., Geyerhofer U., Traxler H., Windisch A., Ulm C., Watzek G. (1999). Blood Supply to the Maxillary Sinus Relevant to Sinus Floor Elevation Procedures. Clin. Oral Implant. Res..

[44] Lundgren S., Andersson S., Sennerby L. (2003). Spontaneous Bone Formation in the Maxillary Sinus after Removal of a Cyst: Coincidence or Consequence?. Clin. Implant. Dent. Relat. Res..

[45] Stricker A., Fleiner J., Stübinger S., Schmelzeisen R., Dard M., Bosshardt D.D. (2015). Bone Loss after Ridge Expansion with or without Reflection of the Periosteum. Clin. Oral Implant. Res..

[46] Troedhan A., Kurrek A., Wainwright M. (2012). Biological Principles and Physiology of Bone Regeneration under the Schneiderian Membrane after Sinus Lift Surgery: A Radiological Study in 14 Patients Treated with the Transcrestal Hydrodynamic Ultrasonic Cavitational Sinus Lift (Intralift). Int. J. Dent..

[47] Dwek J.R. (2010). The Periosteum: What Is It, Where Is It, and What Mimics It in Its Absence?. Skelet. Radiol..

[48] Weng D., Hürzeler M.B., Quiñones C.R., Ohlms A., Caffesse R.G. (2000). Contribution of the Periosteum to Bone Formation in Guided Bone Regeneration. Clin. Oral Implant. Res..

[49] Pinchasov G., Juodzbalys G. (2014). Graft-Free Sinus Augmentation Procedure: A Literature Review. J. Oral Maxillofac. Res.

[50] Walschot L.H., Schreurs B.W., Verdonschot N., Buma P. (2011). The Effect of Impaction and a Bioceramic Coating on Bone Ingrowth in Porous Titanium Particles. Acta Orthop..

[51] Alayan J., Vaquette C., Farah C., Ivanovski S. (2016). A Histomorphometric Assessment of Collagen-Stabilized Anorganic Bovine Bone Mineral in Maxillary Sinus Augmentation—A Prospective Clinical Trial. Clin. Oral Implant. Res..

[52] Alayan J., Vaquette C., Saifzadeh S., Hutmacher D., Ivanovski S. (2016). A Histomorphometric Assessment of Collagen-Stabilized Anorganic Bovine Bone Mineral in Maxillary Sinus Augmentation—A Randomized Controlled Trial in Sheep. Clin. Oral Implant. Res..

[53] Pesce P., Menini M., Canullo L., Khijmatgar S., Modenese L., Gallifante G., Del Fabbro M. (2021). Radiographic and Histomorphometric Evaluation of Biomaterials Used for Lateral Sinus Augmentation: A Systematic Review on the Effect of Residual Bone Height and Vertical Graft Size on New Bone Formation and Graft Shrinkage. J. Clin. Med..

[54] Tadjoedin E.S., De Lange G.L., Holzmann P.J., Kuiper L., Burger E.H. (2000). Histological Observations on Biopsies Harvested Following Sinus Floor Elevation Using a Bioactive Glass Material of Narrow Size Range. Clin. Oral Implant. Res..

[55] Jensen O.T., Shulman L.B., Block M.S., Iacono V.J. (1998). Report of the Sinus Consensus Conference of 1996. Int. J. Oral Maxillofac. Implant..

